# Supplementary material for: Non-volatile optical switch of resistance in photoferroelectric tunnel junctions
Source: Nat Commun. 2021 Jan 15;12:382. doi: 10.1038/s41467-020-20660-9 (PMC7810721; doi:10.1038/s41467-020-20660-9)
Supplement: Supplementary file 1 — Supplementary Information [file 41467_2020_20660_MOESM1_ESM.pdf]

Supplementary information for:

Non-volatile optical switch of resistance in photoferroelectric tunnel junctions

Xiao Long, Huan Tan, Florencio Sánchez, Ignasi Fina,* Josep Fontcuberta*

Contents

Supplementary Note 1: Structural characterization 3

Supplementary Note 2: Electric writing in PFM experiments 3

Supplementary Note 3: Extended PFM characterization I 4

Supplementary Note 4: Extended PFM characterization II 5

Supplementary Note 5. Time dependence of the phase-contrast in PFM 6

Supplementary Note 6: Reproducibility of the measured ER loops in STO//LSMO/BTO/Pt heterostructures 7

Supplementary Note 7: Time dependent current characterization 8

Supplementary Note 8: ER endurance in LSMO/BTO sample 9

Supplementary Note 9: Tunneling current analysis 9

Supplementary Note 10: Thickness dependence of the tunneling current in LSMO/BTO 12

Supplementary Note 11: ER dependence on τ_{write} 12

Supplementary Note 12: ER dependence on τ_{write} in several junctions 13

Supplementary Note 13: PFM characterization under red laser illumination 14

Supplementary Note 14: Photoconductance of the bottom electrode (LSMO) 15

Supplementary Note 15: Resistance drop under illumination 16

Supplementary Note 16: Reproducibility of the measured ER loops in STO//LSMO/STO/BTO/Pt heterostructures 17

Supplementary Note 17: ER endurance in LSMO/BTO sample 17

Supplementary Note 18: Suppression of ionic effect in LSMO/STO/BTO sample 18

Supplementary Note 19. Reproducibility of photoinduced switching 19

Supplementary Note 20: Dependence of the photoinduced switching on illumination conditions. 19

Supplementary Note 21: ER loops protocol 20

Supplementary Note 22, 23 and 24: Dependence of ER and optical suppression of ER on junction area 21

Supplementary Note 25: Leakage subtraction 22
Supplementary References 23

Supplementary Note 1: Structural characterization

Supplementary Fig. 1 shows the X-ray diffraction (XRD) θ-2θ scans for the STO//LSMO/BTO/Pt and STO//LSMO/STO/BTO/Pt samples. The BTO (002) reflection is well-visible in both cases together with the LSMO (002). Traces of reflections of polycrystalline Pt are also observed. The BTO peak broadness results from its thickness, which is approximate 4 nm. Note that the difference in the presence or not of the Pt(111) peak is because in one case the beam was focused on the Pt electrodes and not in the other.

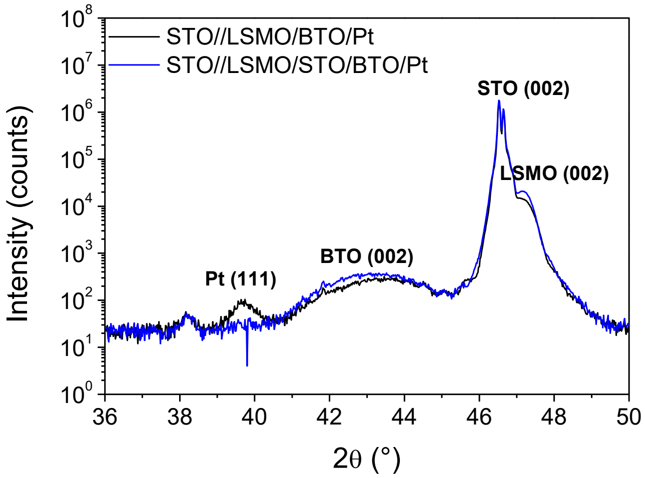

Fig. 1. Structural characterization. XRD θ-2θ scans for the STO//LSMO/BTO/Pt and STO//LSMO/STO/BTO/Pt samples.

Supplementary Note 2: Electric writing in PFM experiments

Supplementary Fig. 2 shows the 10x10 μm region where the positive (+8 V in white) or negative (-8 V in dark) was applied to define regions with P_{DOWN} and P_{UP} states, respectively. The bias voltage was applied while performing a single PFM image.

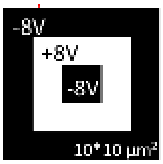

Fig. 2. Electric writing in PFM experiments. The electric writing was done by applying bias voltage (*V* = +8,-8 V for white and black, respectively) at the PFM tip in a region of 10x10 μm^2, while reading in a region of 30x30 μm^2 with zero tip bias. The electric pattern was defined in a single PFM pass.

Supplementary Note 3: Extended PFM characterization I

Supplementary Fig. 3a,b shows PFM amplitude and phase image for written regions in the LSMO/BTO sample, respectively. It can be observed that the phase contrast is 180° and the amplitude is constant across the sample except at the ferroelectric domain walls, as expected for a ferroelectric material. Some regions with lower amplitude signal out from ferroelectric domain walls are observed due to tip bad electrical contact. Equivalent data is shown for the STO/LSMO/BTO sample (Supplementary Fig. 3c,d). It can be observed that both images show contrast with reduced from 180° phase contrast in the phase image. This effect is ascribed to the better insulating properties of the STO/LSMO/BTO sample, which result in greater charging effects which hidden genuine ferroelectric signal as discussed in previous works.[1]

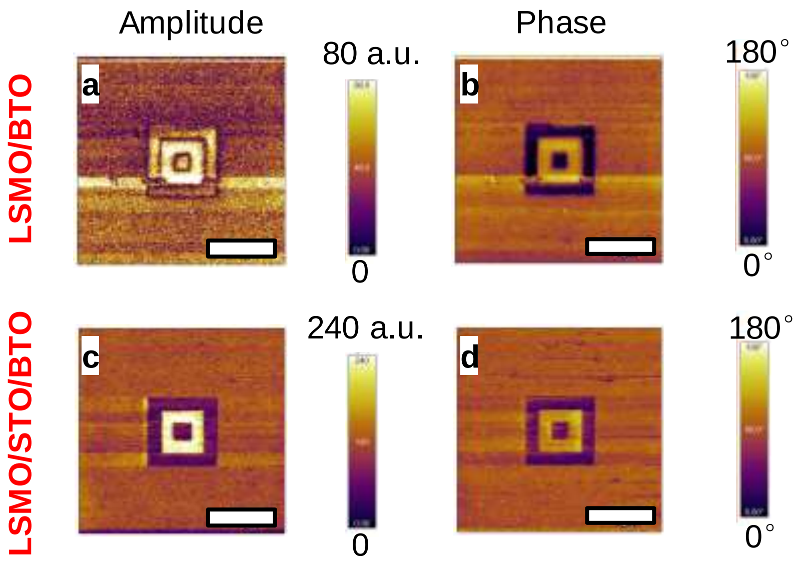

Fig. 3. Charging effects in LSMO/STO/BTO sample. a. amplitude and **b.** phase images of both LSMO/BTO sample. **c.** amplitude and **d.** phase images of LSMO/STO/BTO sample.

Supplementary Note 4: Extended PFM characterization II

Supplementary Fig. 4a shows PFM phase images and profiles collected just after electric writing and after 20 min delay time in dark for the STO//LSMO/BTO sample. The delay time was fix to 20 min, because it is similar to the time between PFM images shown in Fig. 2. It can be observed that the PFM phase contrast is constant among all the collected images/profiles indicating that the polarization retention is high, and disregarding the reduction of polarization contrast shown in Fig. 2 to the action of depolarization fields. Similar set of data is shown in Supplementary Fig. 4b for the STO//LSMO/STO/BTO sample. It can be also observed that the phase contrast is constant comparing the images/profiles collected just after and after waiting 20 min delay time, denoting good retention and disregarding the influence of poor polarization retention in the data shown in Fig.4c. The non-180° phase contrast is discussed in Supplementary Information Supplementary Fig. 3.

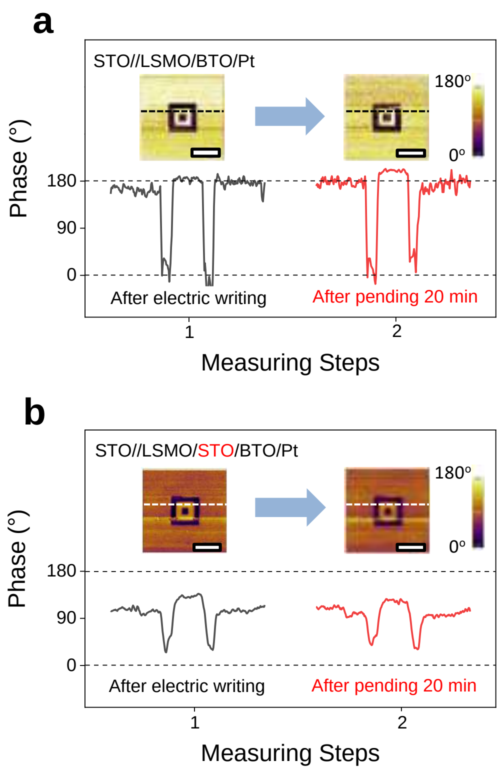

Fig. 4. PFM extended characterization. Out-of-plane domain PFM phase images for both **a.** LSMO/BTO and **b.** LSMO/STO/BTO samples, obtained just after writing P_{DOWN} (inner yellow region) and P_{UP} domains (black region), respectively (left image) and after pending in dark for 20 min (right image). The lines scans shown in the bottom correspond to PFM phase profiles along the dashed lines in the corresponding images for each sample. Scale bar corresponds to 10 μm.

Supplementary Note 5. Time dependence of the phase-contrast in PFM

Supplementary Fig. 5a,b,c show PFM phase images collected after writing with (±8V), either immediately (Original) and after waiting for 1 h and 6 h, respectively. It can be observed that the phase contrast is preserved (Supplementary Fig. 5d) and the dark regions slightly expand with time (Supplementary Fig. 5e) as expected from the presence of E_{imp}.

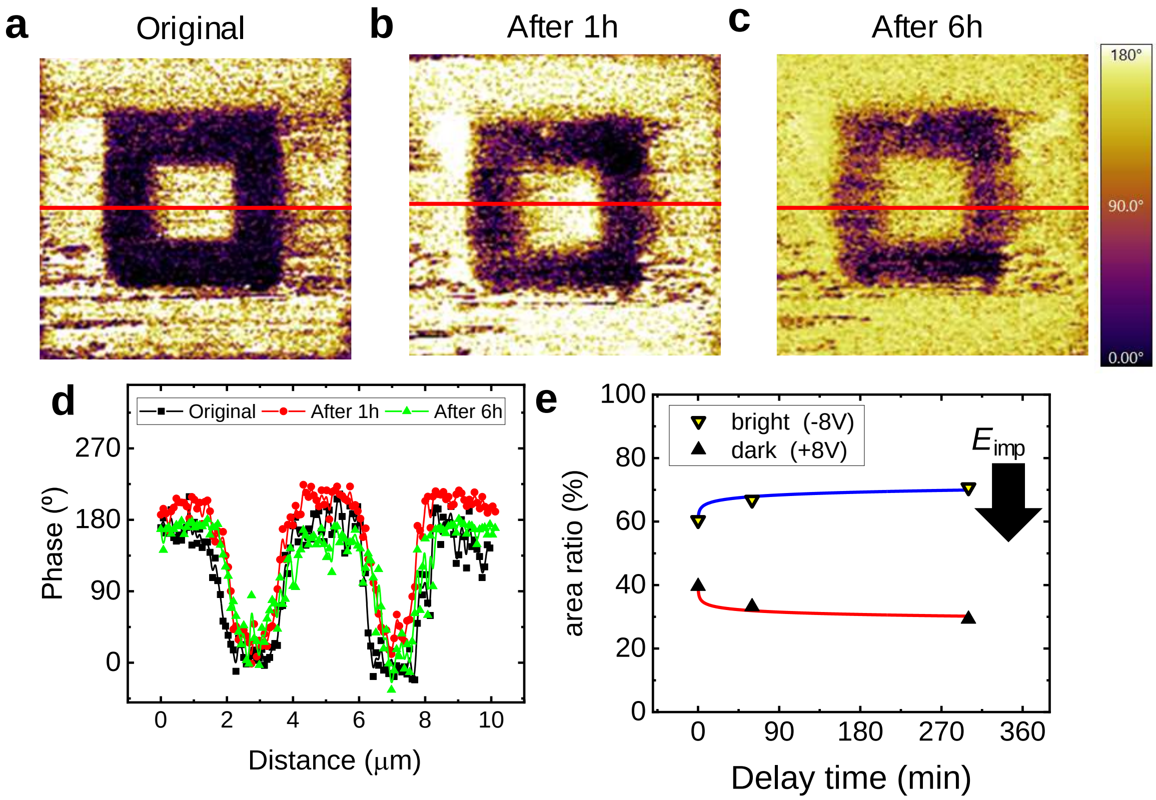

Fig. 5. PFM phase contrast retention. PFM phase images collected after electrical lithography with +8 (bright) and -8V (dark) for the LSMO/BTO sample. **a,b,c.** Data collected freshly after writing (original); after 1 h and after 6 h, respectively. **d.** Line scans (red lines in panels a-c) of the phase-contrast of images in (a,b,c). **e.** Dependence on delay time since electrical writing of relative area ratio of dark/bright areas [=area$_{dark/bright}$/(total area)]. Lines are guides for the eye.

Supplementary Note 6: Reproducibility of the measured ER loops in STO//LSMO/BTO/Pt heterostructures

Supplementary Fig. 6 shows illustrative $R(V_W)$ loop recorded loops recorded using different writing times and in different junctions, in STO//LSMO/BTO/Pt heterostructures. Observation of these loops clearly reveal than the main features are well reproducible during the first cycles.

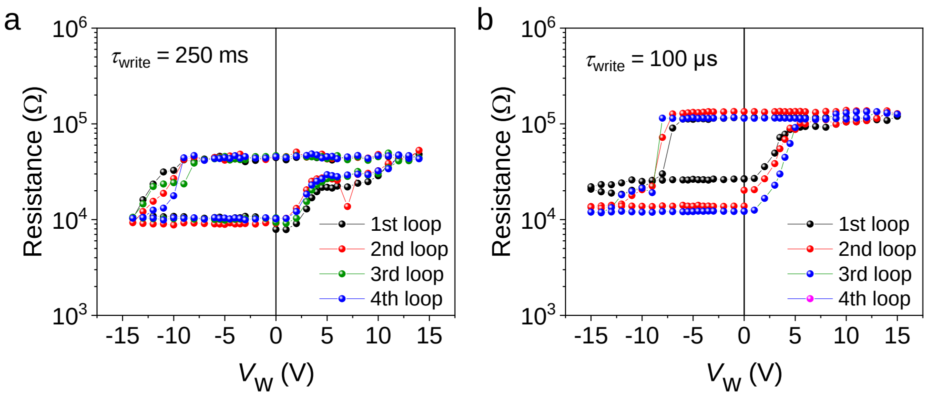

Fig. 6. R(V_W) loop stability. Illustrative examples of the reproducibility of the major ER loops in two different junctions in a STO//LSMO/BTO/Pt sample as a function of the writing time: **a.** 250 ms and **b.** 100 μs. Data show the reproducibility of ER loops over several cycles of operation.

Supplementary Note 7: Time dependent current characterization

Supplementary Fig. 7 shows illustrative Current versus time measurements after prepolarizing the sample with a voltage pulse V_W = -15 V and setting a voltage near $V_{C\text{-LOW}}$ (= 3 V) and $V_{C\text{-HIGH}}$ (= 10 V) in the LMSO/BTO sample. It can be observed that the current flowing under V = 3V is nearly constant. Instead, the current flowing under V = 10V shows a gradual reduction, with a time constant of about $\approx$ 1 s. Although, the time scale of the latter result might be compatible with soft breakdown, we should emphasize that we observe a reduction of current, that is an increase of the device resistance, which is opposite to the resistance decrease observed when SBD occurs in $BaTiO_3$.[2]

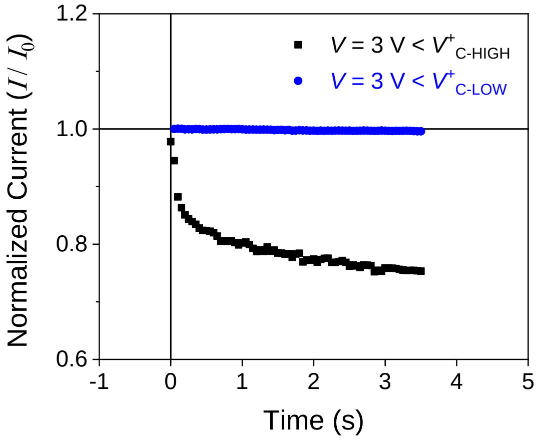

Fig. 7. Time dependent current experiments. Time dependence of the normalized current for applied voltages near $V_{C\text{-LOW}}$ (= 3 V) and $V_{C\text{-HIGH}}$ (= 10 V) for the LSMO/BTO sample. Zoom of the time dependence of the normalized current for applied voltages near $V_{C\text{-LOW}}$ (= 3 V).

Supplementary Note 8: ER endurance in LSMO/BTO sample

HRS and LRS states obtained sequentially by the application of hundreds of ±8V pulses ($V_{C\text{-}LOW}$ > 8V < $V_{C\text{-}HIGH}$) are shown for the LSMO/BTO sample in Supplementary Fig. 8a. As shown in the manuscript, at this rather voltage, ionic conduction contribution is of little relevance for ER. The data collected for up to 200 cycles demonstrate that the two resistance states remain relatively constant and are well distinguishable. In other words, the device can be robustly switched. The observed variations of resistance can be ascribed to electrical noise and /mechanical instabilities. In Supplementary Fig. 8b, HRS and LRS states obtained sequentially by the application of much larger voltage pulses (±15V) are shown. Notice that V = +15 V is well above $V_{C\text{-}HIGH}$, where ionic motion is predominant. We observe that the junction degrades fast, after 20 cycles for BTO. This fast degradation is in agreement with the proposed ionic conduction mechanism but, a soft dielectric breakdown after several cycles, cannot be excluded.

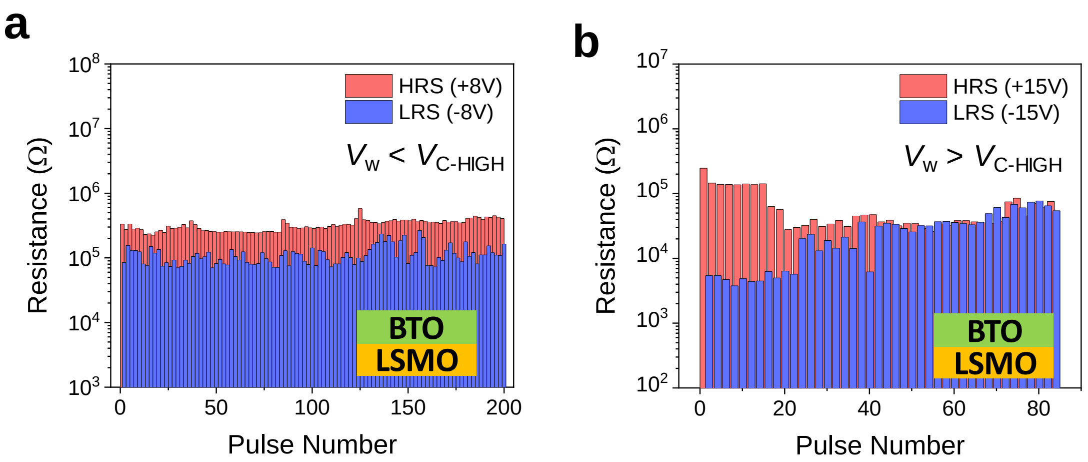

Fig. 8. Endurance for LSMO/BTO sample. a. Endurance of HRS states and LRS states written by ±8 V in a junction of STO//LSMO/BTO/Pt sample. **b.** Endurance of HRS states and LRS states written by ±15 V in a junction of STO//LSMO/BTO/Pt sample.

Supplementary Note 9: Tunneling current analysis

Supplementary Fig. 9a shows $I(V)$ characteristics in the low-voltage region (0.5 V) for the indicated LRS (written either by -8 or -15 V), HRS (V_W = +8 V) and HRS^{ION} (V_W = +15 V) states

and data fitting. The fittings are obtained by minimizing the χ^2 value accounting for the difference between the experimental values and the fitted ones [χ^2=Σ(experimental-fitted)^2] using the equation:[3, 4]

$$J \cong C \frac{\exp\left\{\alpha(V)\left[\left(\Phi_2-\frac{eV}{2}\right)^{3/2}-\left(\Phi_1+\frac{eV}{2}\right)^{3/2}\right]\right\}}{\alpha^2(V)\left[\left(\Phi_2-\frac{eV}{2}\right)^{1/2}-\left(\Phi_1+\frac{eV}{2}\right)^{1/2}\right]^2} \times \sinh\left\{\frac{3}{2}\alpha(V)\left[\left(\Phi_2-\frac{eV}{2}\right)^{1/2}-\left(\Phi_1+\frac{eV}{2}\right)^{1/2}\right]\frac{eV}{2}\right\}$$

where

$$C = -(4em^*m_e)/(9\pi^2\hbar^3)$$
$$\alpha(V) \equiv [4d(2m^*m_e)^{1/2}]/[3\hbar(\Phi_1 + eV - \Phi_2)]$$

and m_e corresponds to the electron mass, m^* to its effective mass (fixed to 1), e to its charge, d to the tunneling thickness and Φ_1 and Φ_2 corresponding to the LSMO/BTO and BTO/Pt interfaces, respectively.

In Supplementary Fig. 9b it is sketched the band alignment of isolated materials (values are obtained according to refs. [5-8]) and in Supplementary Fig. 9c the band alignment of the junction. The values obtained are similar to those obtained by the described data fitting, which are summarized in Supplementary Table 1. It can be observed that the fitted values of Φ_1 (0.5-0.6 eV) and Φ_2 (1.4-1.8 eV) corresponding to the LSMO/BTO and BTO/LSMO interfaces, respectively are in agreement with those obtained from band alignment analysis Φ_1 = 1 eV and Φ_2 = 1.8 eV. The discrepancy is attributed to the high modulation of d parameter, accounting for tunneling thickness, which dominates in the shape of the fitted

$I(V)$ curves. Overall data in Supplementary Fig. 9 indicates tunneling transport across the film thickness.

Attempts to fit $I(V)$ characteristics using Fowler-Nordheim tunneling (FNT) and Thermionic injection (TI) following the equations described in Pantel et al.[9] It turns out that good fits can be obtained by using the TI model and the extracted barrier energies are almost identical ($\approx$ 0.5 V) for both barrier sides. A barrier of $\approx$ 0.5 V for the BTO/Pt interface is much smaller than that expected from simple estimates of the barrier height on the basis of work function of Pt and electron affinity of BTO. We had therefore concluded that the fits using FNT and TI models were not meaningful.

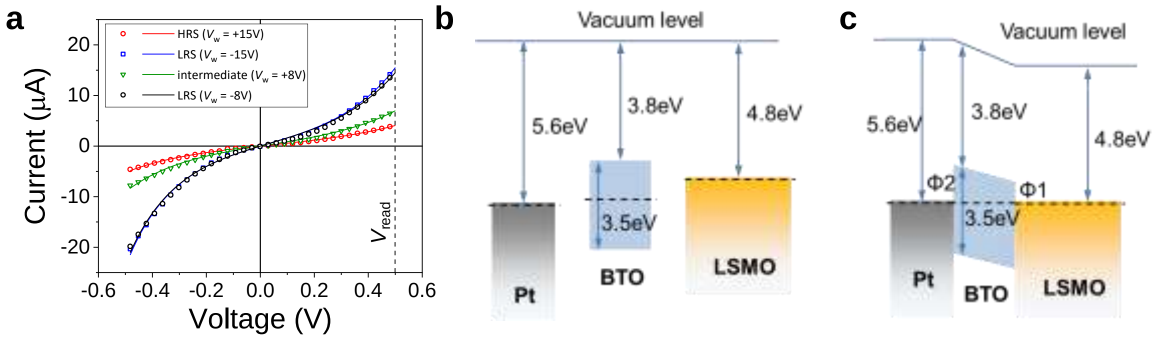

Fig. 9. Tunneling current and barrier analysis. a. $I(V)$ characteristics measured at room temperature on BTO junction of 4 nm. The $I(V)$ curves were measured for different resistance state: HRS^{ION} (V_W = +15 V), LRS (V_W = -15 V, -8 V), and HRS (V_W = +8 V). All the τ_{write} used here was constantly 1ms. **b.** Sketch of the band structure before contact. **c.** Sketch of band structure in the equilibrium state after contact.

Table 1. Fitting parameters obtained from $I(V)$ curves in Supplementary Fig. 9a.

V_W (V)	Φ_1 (eV)	Φ_2 (eV)	d (nm)
-15 V	0.5	1.5	4.7
-8 V	0.5	1.4	4.7
+8 V	0.5	1.4	5.2
+15 V	0.6	1.8	4.9

Supplementary Note 10: Thickness dependence of the tunneling current in LSMO/BTO

Supplementary Fig. 10 shows the resistance of junctions of several samples of different thickness grown in nominally the same conditions to that reported in the main text and its dependence of the polarization direction dictated by writing pulses of V_W = ± 5 V. It can be appreciated that, for both polarization states (up and down triangles), the resistance increases roughly exponentially with thickness. We also include the resistance values at the HRS obtained by suitable illumination (circles). The coincidence of HRS obtained by voltage pulses and illumination is remarkable.

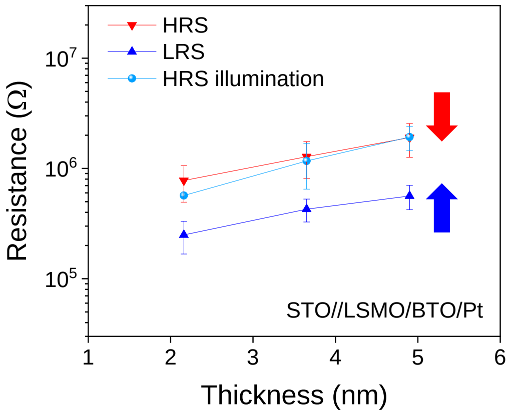

Fig. 10. Resistance of samples of different thickness. Resistance dependence on BTO thickness of the voltage (downward red triangle) and light (blue circles) written HRS states and LRS (upward blue triangle) state in STO//LSMO/BTO/Pt sample. Error bars correspond to standard deviation of 5 measurements.

Supplementary Note 11: ER dependence on τ_{write}

Supplementary Fig. 11a-e shows the $R(V_W)$ loops collected for different τ_{write}. In Supplementary Fig. 11f, LRS, HRS and HRS^{ION} states, corresponding to resistance measured after V_W = -15, +8 and +15 V, respectively, dependence on τ_{write} is plotted. It can be observed that the two resistance states LRS and HRS are visible irrespective of τ_{write}, while the HRS^{ION} only appears at τ_{write} > 500 μs.

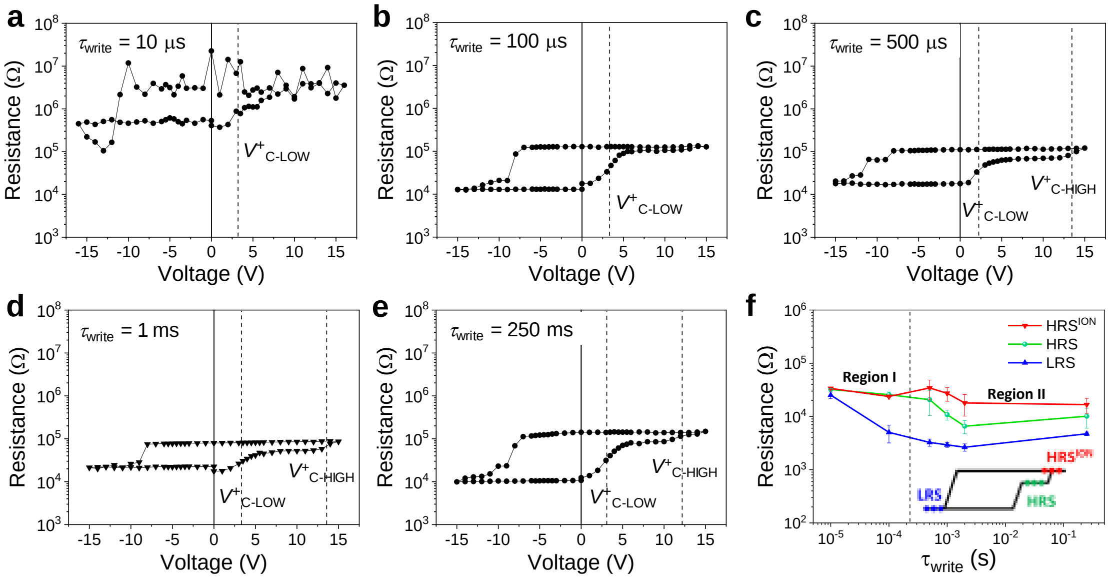

Fig. 11. ER dependence on τ_{write}. $R(V_W)$ loops (from -15V to 15V) were collected in a series of writing time (τ_{write} from 10^{-5}s to 0.25s), in the LSMO/BTO sample: **a-e**. **f.** Dependence of of HRS^{ION} (red), LRS (blue) and HRS (green) on τ_{write}. The plot is divided into two regions depending on whether HRS^{ION} is absent or not. Error bars correspond to standard deviation of 5 measurements.

Supplementary Note 12: ER dependence on τ_{write} in several junctions

Supplementary Fig. 12 shows ER dependence on τ_{write} in four junctions, which show similar behavior of data shown in Supplementary Fig. 11f, although some variability in the absolute resistance values is observed.

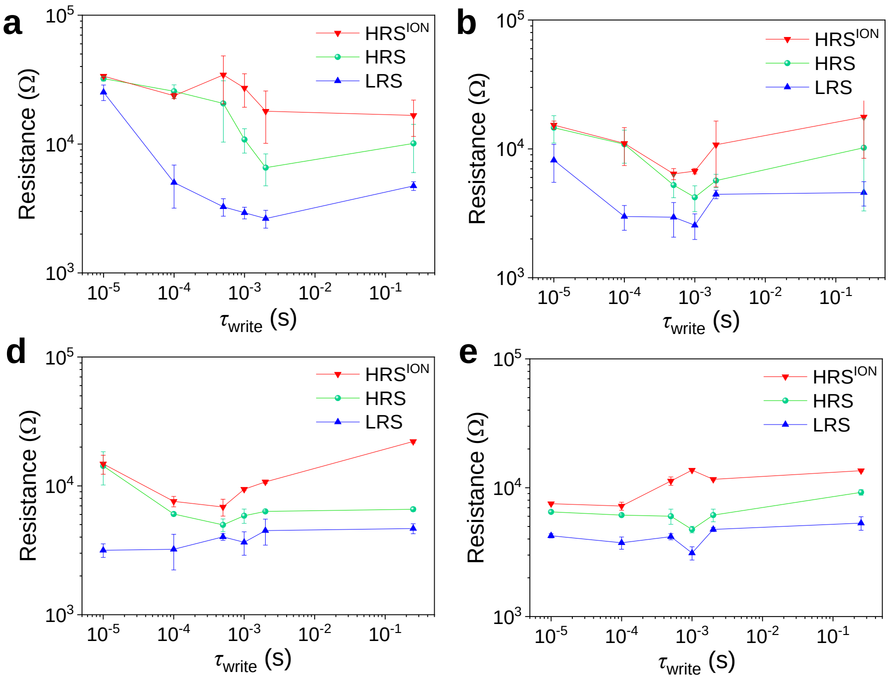

Fig. 12. ER dependence on τ_{write} in several junctions. Dependence of different resistance state (HRS^{ION}, red; LRS, blue; HRS, green) on writing time recorded in 4 junctions. Error bars correspond to standard deviation of 5 measurements.

Supplementary Note 13: PFM characterization under red laser illumination

In Supplementary Fig. 13a,b,c, PFM-phase images just after, 10 min after and after 10 min of red (λ = 638 nm, E_ν = 1.94 eV, 48.5 W/cm^2) laser illumination after electrical writing, respectively, are shown. No change is observed.

LSMO is expected to absorb red light due to its small bandgap of 0.6 eV.[10] Instead, BTO is not expected to not absorb light due to its greater bandgap (Eg = 3.3 eV),[11] much above E_ν = 1.94 eV. Thus, if LSMO has a relevant role in the polarization switching under blue light illumination, the polarization switching should be reproducible under red illumination. Instead, if BTO has a relevant role in the polarization switching under blue illumination, the polarization switching is expected to not be reproducible under red illumination. Therefore, the results summarized in Supplementary Fig. 13a,b,c disregard important contribution from LSMO bottom electrode in the polarization

switching under blue illumination. The results shown in Supplementary Fig. 13 also disregard important contribution of thermal effects, which should be similar for red and blue illumination.

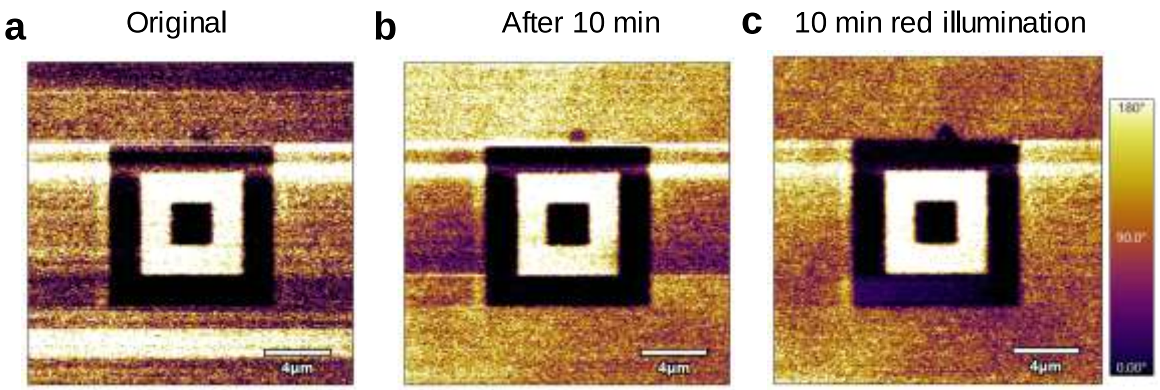

Fig. 13. PFM phase contrast under red illumination. a. PFM phase images collected just after electrical lithography with +8 (bright) and -8 V (dark). **b.** PFM phase images collected 10 min after electrical lithography with +8 (bright) and -8 V (dark). for the LSMO/BTO sample. **c.** PFM phase images collected after 10 min illumination with red light red (λ = 638 nm, E_{ν} = 1.94 eV, 48.5 W/cm^2) after electrical lithography with +8 (bright) and -8V (dark).

Supplementary Note 14: Photoconductance of the bottom electrode (LSMO)

We have measured the dependence of the resistivity of LSMO on illumination by using the same conditions (same power and wavelength) than used in the experiments reported in the manuscript. Using a 4-probe configuration, the resistance of the bottom layer in dark and under illumination has been recorded. In Supplementary Fig. 14, below we show the results. The resistivity is ≈ 1 mΩ·cm in agreement in literature data for similar films. Any photoresistance in our films is below noise level (<< 1m·Ωcm). This observation excludes a contribution of LSMO photoconductance to the resistance changes observed in the manuscript.

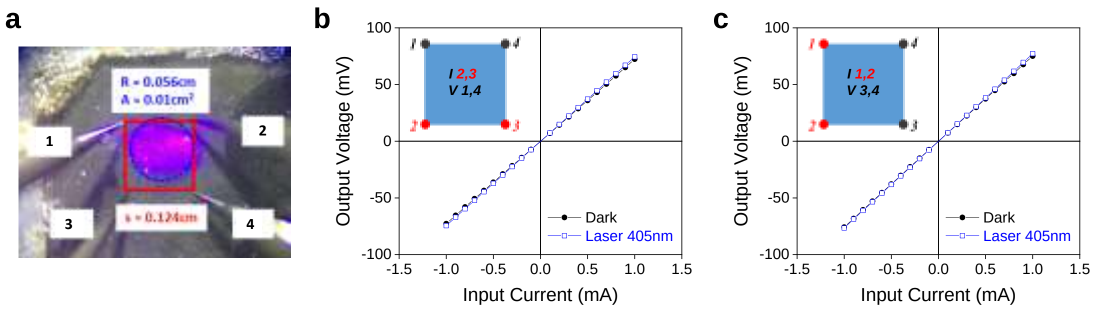

Fig. 14. a. Experimental set up to measure the LSMO film resistivity in the LSMO/BTO sample. Four needles adjacent to an array of 4 neighboring 4 Pt are used to measure in 4 probe configuration the LSMO resistivity. **b,c.** *I*(*V*) measurements recorded in two different configurations, obtained by permuting current and voltage probes. Date recorded in dark (black symbols) and under illumination (blue symbols) are shown in (b) and (c).

Supplementary Note 15: Resistance drop under illumination

Supplementary Fig. 15 shows $R(V_W)$ loops measured sequentially in dark (black solid squares), under illumination (blue solid circles) and after illumination (black solid squares) in 3 different junctions of STO//LSMO/BTO/Pt sample. It can be observed that the loop collected under illumination always show less contrast that in dark conditions irrespectively if the loop is collected before or after illumination. It can be also observed that the loop measured in dark after illumination is not the same than the one measured before, indicating some irreversibility during the illumination process discussed in the main text.

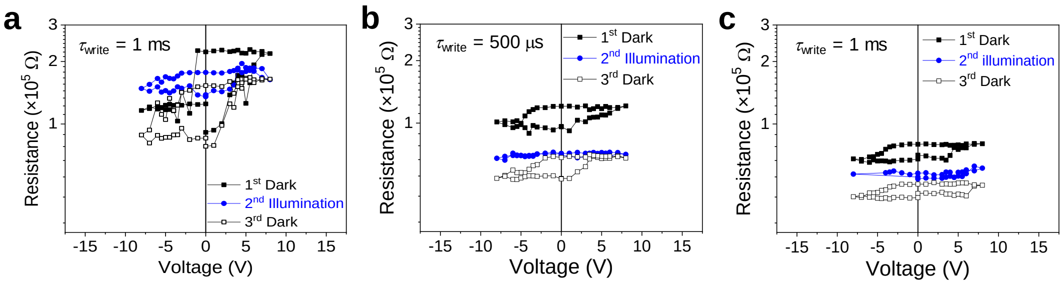

Supplementary Fig. 15. Non-reversible photoresponse. $R(V_W)$ loops collected (-8V ~ 8V) in LSMO/BTO samples following the sequence: dark → illumination → dark.

Supplementary Note 16: Reproducibility of the measured ER loops in STO//LSMO/STO/BTO/Pt heterostructures

Supplementary Fig. 16 shows illustrative $R(V_W)$ loop recorded loops recorded using different writing times and in different junctions, in STO//LSMO/STO/BTO/Pt heterostructures. Observation of these loops clearly reveal than the main features are well reproducible.

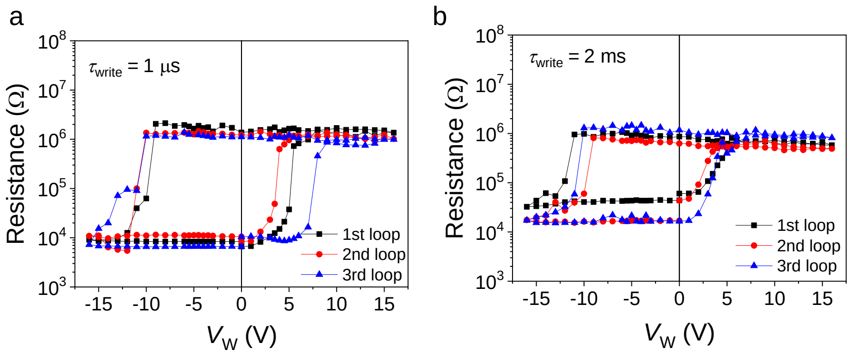

Fig. 16. $R(V_W)$ loop reproducibility. Illustrative examples of the reproducibility of the major ER loops in two different junctions in a STO//LSMO/STO/BTO/Pt sample for different writing time: **a.** 1 μs, **b.** 10 μs, **c.** 2 ms and **d.** 250 ms. Data show the reproducibility of ER loops over several cycles of operation.

Supplementary Note 17: ER endurance in LSMO/BTO sample

HRS and LRS states obtained sequentially by the application of hundreds of ±8V pulses ($V_{C\text{-}LOW}$ < 8V < $V_{C\text{-}HIGH}$) are shown for the LSMO/STO/BTO sample in Supplementary Fig. 17. As shown in the manuscript, at this rather voltage, ionic conduction contribution is negligible for ER. The data collected for up to 200 cycles demonstrate that the two resistance states remain constant and are well distinguishable. In other words, the device can be robustly switched. The observed variations of resistance can be ascribed to electrical noise and /mechanical instabilities.

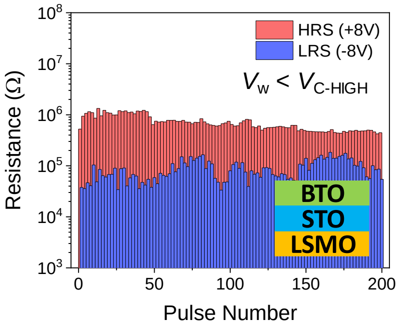

Fig. 17. Endurance for LSMO/BTO sample. Endurance of HRS states and LRS states written by ±8 V in a junction of STO//LSMO/STO/BTO/Pt sample.

Supplementary Note 18: Suppression of ionic effect in LSMO/STO/BTO sample

Supplementary Fig. 18 shows $R(V_W)$ loops collected for indicated τ_{write} for the LSMO/STO/BTO sample. It can be observed that the $V_{C\text{-LOW}}$ is well-visible, whereas the $V_{C\text{-HIGH}}$ is not, indicating the suppression of the HRS^{ION} state. This indicates that the introduction of the STO dielectric layer contributes to mitigate the ionic effect.

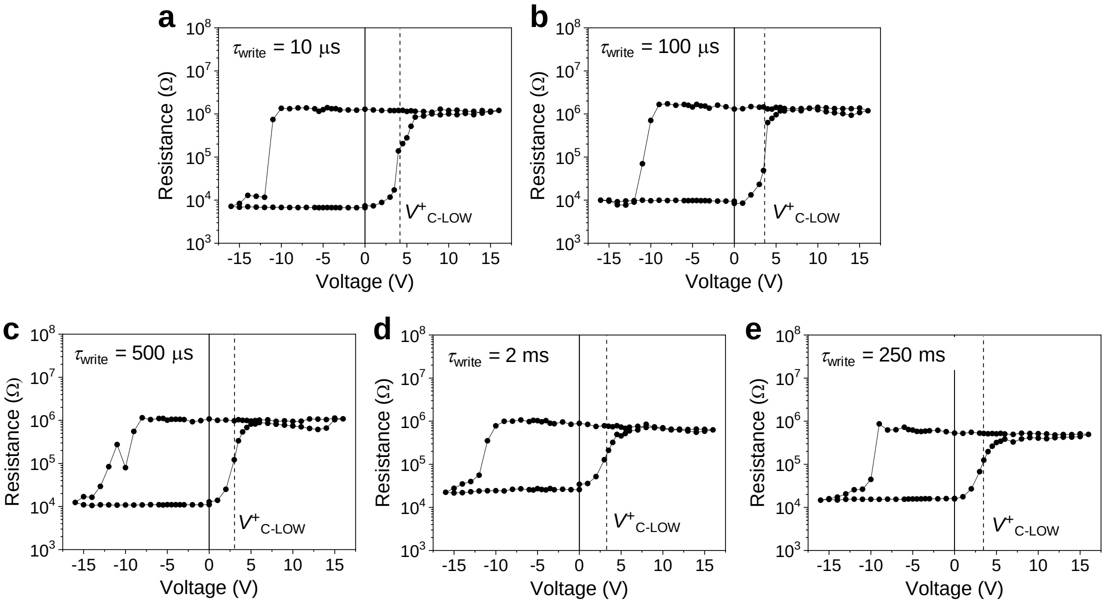

Fig. 18. Suppression of ionic effect in LSMO/STO/BTO sample. a-e. $R(V_W)$ loops collected for indicated τ_{write} for the LSMO/STO/BTO sample.

Supplementary Note 19. Reproducibility of photoinduced switching

In Supplementary Figure 19, we depict the resistance obtained after electrical and optical writing for several junctions. Although, as mentioned in the main manuscript, absolute resistance values display some sample-to-sample variability, the optical switch of resistance is systematic observed.

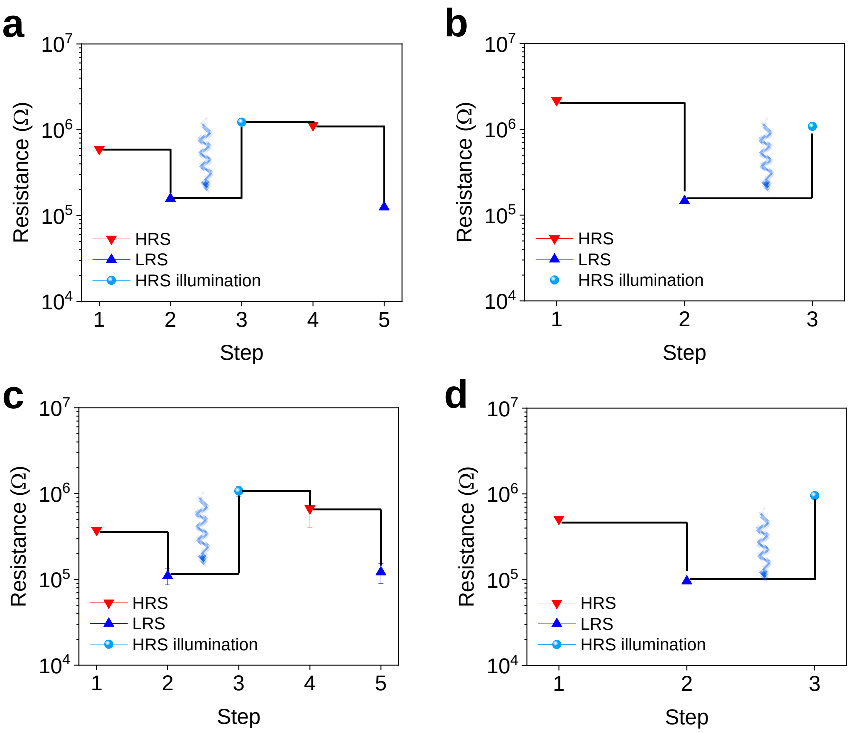

Fig. 19. Optical switch stability for different junctions. a,b,c,d. Resistance states obtained sequentially after electrical and optical stimuli (as indicated) for several junctions, respectively. In this example: $V_W = \pm 8$ V. Illumination is performed. Illumination is done using λ = 405 nm and power density of 48.5 W/cm^2.

Supplementary Note 20: Dependence of the photoinduced switching on illumination conditions.

Experiments have been conducted to determine how the resistance of the HRS state induced by light depends on illumination conditions (power and duration of light pulses). Results, shown in Supplementary Fig. 20 below, indicate that illumination produces a gradual switching from LRS to HRS, which timescale is dictated by the power of the laser and the duration of the illumination step.

We also include in this plot the resistance of the HRS and LRS, written in dark. It is remarkable the close coincidence of the final states (HRS) obtained by V-pulses and light-pulses.

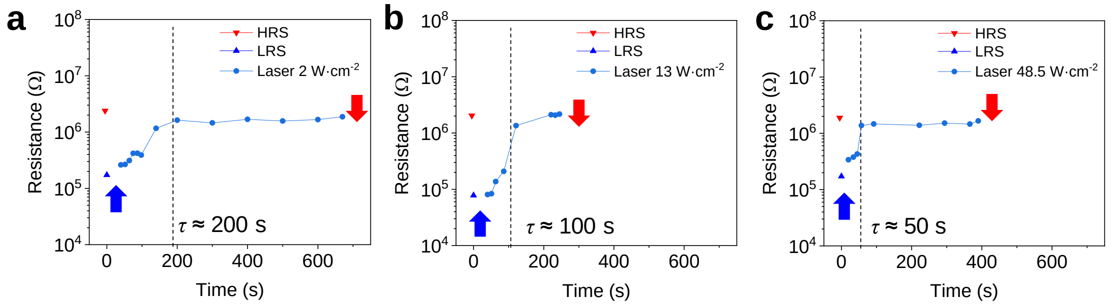

Fig. 20. **Evolution of the resistance of the junctions under illumination from LRS to HRS. a,b,c.** Restistance data (blue circles) collected as function of illumination time when different laser power are used (2, 13, and 48.5 W/cm^2, respectively). Each plot includes the resistance of the LRS and HRS measured in dark (up blue and down red, respectively).

Supplementary Note 21: ER loops protocol

Supplementary Fig. 21a shows the schematics to measure *I*(*V*) characteristics after application of V_W writing pulses. Positive (red) or negative (blue) trapezoidal signals of total duration τ_{write} are applyied. Afterwards, *I*(*V*) characteristics is measured after 1 s delay time of voltage pulse in a 0.5 V voltage range. The $R(V_W)$ loop is measured repeating the protocol showed in Supplementary Fig. 21a for increasing and decreasing V_W, as shown in Supplementary Fig. 21b.

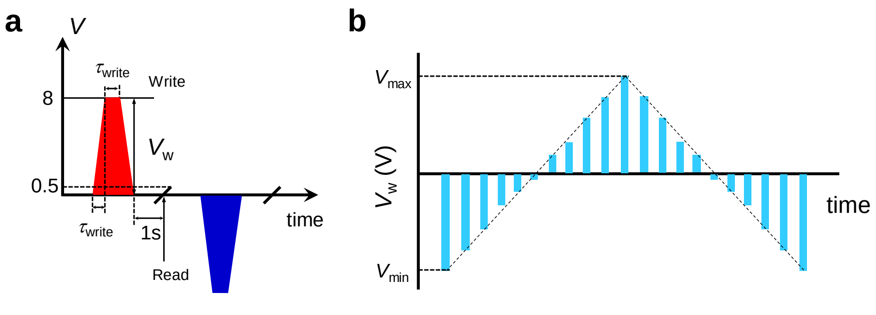

Fig. 21. ER loops protocol. a. Sketch of the *I*(*V*) measuring protocol, for writing and reading junction resistance. Trapezoid writing pulse is applied before measuring *I*(*V*) at maximum applied voltage of 0.5 V, and the delay time is constantly fixed to 1 s for all the experiments. **b.** Sketch of the ER measurement sequence following the V_{min} to V_{max} to V_{min} path (for instance, V_{min} = -8V, V_{max} = +8V).

Supplementary Note 22, 23 and 24: Dependence of ER and optical suppression of ER on junction area

In Supplementary Fig. 22, we show data for STO//LSMO/STO/BTO/Pt. The switching response is clearly observed using both electrodes (Fig, 22a) and in the corresponding ER loops (Supplementary Fig. 22b). In Supplementary Fig. 23, additional data collected in different junctions of 7 μm size in STO//LSMO/STO/BTO/Pt hetersostructure is shown. Data corresponding to the junctions of 20 μm size shown in Supplementary Fig. 22b is also included for comparison. In data in Supplementary Fig. 22 and 23 it can be appreciated that junction resistance and the ER are larger in the smaller junctions, as commonly found. This is typically related to the existence of non-switchable low-resistance channels in the barriers. The fact that the effect is area-dependent disregards filamentary conduction as triggering mechanism of the found effect. In Supplementary Fig. 24, the ER suppression under illumination for electrodes of different sizes is shown. It can be observed that the suppression under illumination is robust, irrespectively of the electrode size.

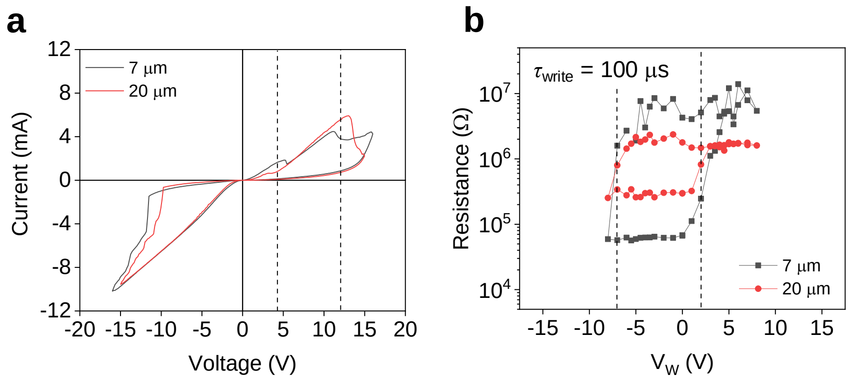

Fig. 22. Dependence of ER on electrode area for STO/BTO sample. a. Illustrative examples of the I(V) curves and **b.** ER recorded in dark in junctions in STO//LSMO/STO/BTO/Pt having electrodes with diameter of 7 μm and 20 μm, as indicated.

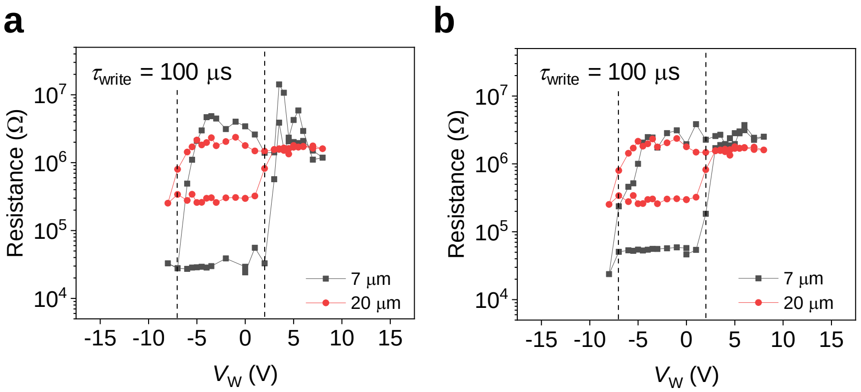

Fig. 23. Dependence of ER on electrode area for STO/BTO sample. a,b. Illustrative ER loops recorded in dark in different junctions, respectively, in STO//LSMO/STO/BTO/Pt having electrodes with diameter of 7 μm, as indicated. Reference loop obtained in the representative 20 μm junction of Fig. 22b is also included in both panels.

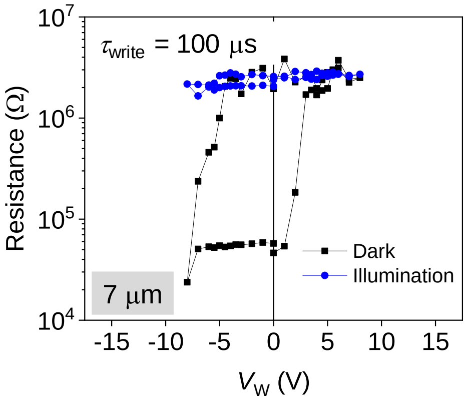

Fig. 24. Dependence of optical switch on electrode area for STO/BTO sample. Illustrative ER loops recorded in dark and under illumination in electrodes with diameter of 7 μm of STO//LSMO/STO/BTO/Pt sample.

Supplementary Note 25: Leakage subtraction

In Supplementary Fig. 25, we show ferroelectric characterization performed on both LSMO/BTO and LSMO/STO/BTO samples. $I(V)$ characteristics collected at 5 kHz in top-top configuration show important leakage contribution. Superimposed to it clear ferroelectric current switching peaks are observed (enclosed by circles). Its contribution has been isolated by fitting the leakage contribution to an exponential dependence and subtracting it from the raw data. Free of leakage $I(V)$ and $P(V)$ loops shown in Figs. 3d and 4d for the LSMO/BTO and LSMO/STO/BTO samples, respectively are obtained. The reasonable polarization values obtained indicate that ferroelectric nature of the observed current switching peaks.

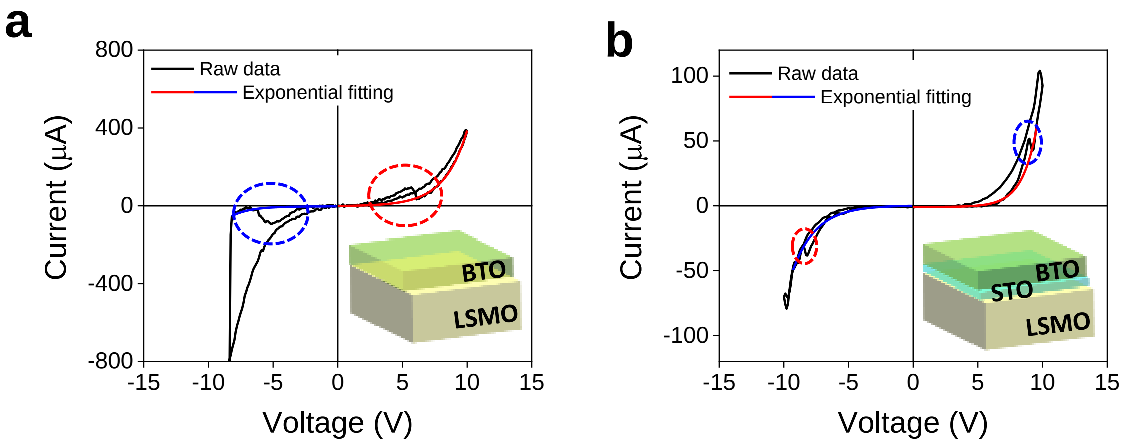

Fig. 25. Leakage subtraction. a. Illustrative examples of the *I*(*V*) curves collected at 5 kHz for the LSMO/BTO sample. The leakage contribution has been fitted by exponential curve. This contribution has been removed from the *I*(*V*) characteristics and the subtracted curve is shown in Fig. 3d. **b.** Equivalent to (a) data for the LSMO/STO/BTO sample. The subtracted curve is shown in Fig. 4d.

Supplementary References

1. Kim S., Seol D., Lu X., Alexe M., Kim Y. Electrostatic-free piezoresponse force microscopy. *Sci. Rep.* **7**, 41657 (2017).
2. Desu S. B., Yoo I. K. Time-Dependent Dielectric Breakdown in $BaTiO_3$ Thin Films. *J. Electrochem. Soc.* **140**, L133 (1993).
3. Gruverman A.*, et al.* Tunneling electroresistance effect in ferroelectric tunnel junctions at the nanoscale. *Nano Lett.* **9**, 3539-3543 (2009).
4. Brinkman W., Dynes R., Rowell J. Tunneling conductance of asymmetrical barriers. *J. Appl. Phys.* **41**, 1915-1921 (2003).
5. Cardona M. Optical Properties and Band Structure of $SrTiO_3$ and $BaTiO_3$. *Phys. Rev.* **140**, A651 (1965).
6. Piskunov S., Heifets E., Eglitis R., Borstel G. Bulk properties and electronic structure of $SrTiO_3$, $BaTiO_3$, $PbTiO_3$ perovskites: an ab initio HF/DFT study. *Computat*IONal *Mat*ER*ials Science* **29**, 165-178 (2004).
7. Reagor D., Lee S., Li Y., Jia Q. Work function of the mixed-valent manganese perovskites. *J. Appl. Phys.* **95**, 7971-7975 (2004).
8. Hölzl J., Schulte F. K., Wagner H. *Solid surface physics*. Springer-Verlag Berlin Heidelberg New York, Germany (2006).
9. Pantel D., Alexe M. Electroresistance effects in ferroelectric tunnel barriers. *Phys. Rev. B* **82**, 134105 (2010).
10. Lee H.-S., Park H.-H. Band structure analysis of $La_{0.7}Sr_{0.3}MnO_3$ perovskite manganite using a synchrotron. *Advances in Condensed Matt*ER *Physics* **2015**, 746475 (2015).
11. Wemple S. Polarization Fluctuations and the Optical-Absorption Edge in $BaTiO_3$. *Phys. Rev. B* **2**, 2679 (1970).
